# Inhibitory Effect of Selaginellins from *Selaginella tamariscina* (Beauv.) Spring against Cytochrome P450 and Uridine 5′-Diphosphoglucuronosyltransferase Isoforms on Human Liver Microsomes

**DOI:** 10.3390/molecules22101590

**Published:** 2017-09-21

**Authors:** Jae-Kyung Heo, Phi-Hung Nguyen, Won Cheol Kim, Nguyen Minh Phuc, Kwang-Hyeon Liu

**Affiliations:** 1 BK21 Plus KNU Multi-Omics Based Creative Drug Research Team, College of Pharmacy and Research Institute of Pharmaceutical Sciences, Kyungpook National University, 80 Daehakro, Bukgu, Daegu 41566, Korea; anna4602@gmail.com (J.-K.H.); wk3012@naver.com (W.C.K.); phucnguyen0606@gmail.com (N.M.P.); 2 Institute of Natural Products Chemistry, Vietnam Academy of Science and Technology, 18-Hoang Quoc Viet, Cau Giay, Hanoi 122100, Vietnam; nguyenphihung1002@gmail.com

**Keywords:** cytochrome P450, drug interaction, mass spectrometry, selaginellins, uridine 5′-diphosphoglucuronosyltransferase

## Abstract

*Selaginella tamariscina* (Beauv.) has been used for traditional herbal medicine for treatment of cancer, hepatitis, and diabetes in the Orient. Numerous bioactive compounds including alkaloids, flavonoids, lignans, and selaginellins have been identified in this medicinal plant. Among them, selaginellins having a quinone methide unit and an alkylphenol moiety have been known to possess anticancer, antidiabetic, and neuroprotective activity. Although there have been studies on the biological activities of selaginellins, their modulatory potential of cytochrome P450 (P450) and uridine 5′-diphosphoglucuronosyltransferase (UGT) activities have not been previously evaluated. In this study, we investigated the drug interaction potential of two selaginellins on ten P450 isoforms (CYP1A2, 2A6, 2B6, 2C8, 2C9, 2C19, 2D6, 2E1, 2J2 and 3A) and six UGT isoforms (UGT1A1, 1A3, 1A4, 1A6, 1A9 and 2B7) using human liver microsomes and liquid chromatography-tandem mass spectrometry. Selaginellin and selaginellin M had high inhibitory potential for CYP2C8-mediated amodiaquine *O*-demethylation with IC_50_ values of 0.5 and 0.9 μM, respectively. Selaginellin and selaginellin M also showed medium inhibitory potential against CYP2C9, CYP2J2, UGT1A1, and UGT1A3 (1 μM < IC_50_ < 5 μM). These two selaginellins had low inhibitory potential against CYP1A2, CYP2A6, CYP2E1, and UGT1A6 (IC_50_ > 25 μM). This information might be helpful to predict possible drug interaction potential of between selaginellins and co-administered drugs.

## 1. Introduction

*Selaginella tamariscina* (Beauv.) which belongs to Selaginellaceae, has been traditionally used in treating blood in excrement, hematuria, inflammation, chronic hepatitis, and hyperglycemia in the Orient, especially in China [1,2]. A number of alkaloids, flavonoids, lignans, selaginellins, phenolics, and terpenoids were reported as chemical constituents of *S. tamariscina* [3]. Among these constituents, selaginellins are another group of polyphenolics with a p-quinone methide unit and an alkynylphenol carbon skeleton [4]. Pharmacological studies demonstrate that selaginellins have been known to have anticancer [5,6,7], antidiabetic [8,9], antimicrobial [10,11], antioxidant [12,13], antihyperlipidemic [13], and neuroprotective [14] activities.

Use of botanical drugs to prevent common disease is on the rise among the global population [15]. Since botanical drugs share the same drug metabolizing enzymes with commonly used commercial drugs, the potential for herb–drug interaction is substantial [16]. Several medicinal herbs and foods, including St. John’s wort [17] and grapefruit juice [18] as well as their active constituents (hyperforin [19] and bergamottin [20]) have been reported to cause severe drug interactions. Undoubtedly, the early evaluation of herb–drug interactions is necessary to prevent potential dangerous clinical outcomes.

Modulation of drug-metabolizing enzymes is one of the important causes of drug–drug or herb–drug interaction. Among the numerous drug-metabolizing enzymes, cytochrome P450s (P450s) and uridine 5′-diphosphoglucuronosyltransferases (UGTs), which are responsible for the metabolic clearance of 90% of commercial drugs, have been shown to a play key roles in drug metabolism and drug interactions [21]. For example, bergamottin is reported to increase the blood concentration of drugs through inhibition of hepatic CYP3A activity, thereby enhancing the toxicity of drugs such as atorvastatin, felodipine, and verapamil [22]. Pre-treatment with psoralidin, which has inhibitory potential against UGT1A1-mediated SN-38 glucuronidation (Ki = 5.8 μM), was shown to increase the toxicity of irinotecan [23]. Accordingly, P450- and UGT-mediated drug interactions are even more critical.

**Figure 1:**
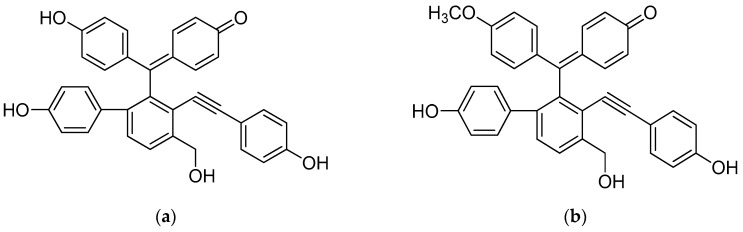
Chemical structures of selaginellin and selaginellin M from *S. tamariscina*: (**a**) Selaginellin; (**b**) Selaginellin M.

Therefore, modulation of selaginellins on P450 and UGT activities may result in potential increase of the systemic exposures of co-administered drugs. To the best of our knowledge, however, no previous study has reported the modulatory effects of selaginellins against human P450 and UGT activities. Here, we investigated the inhibitory potential of two selaginellins (Figure 1) on ten P450- and six UGT-isoform activities in human liver microsomes (HLMs) using cocktails of P450 or UGT probe substrates to evaluate the possibility of drug interactions of selaginellins.

## 2. Results and Discussion

**Figure 2:**
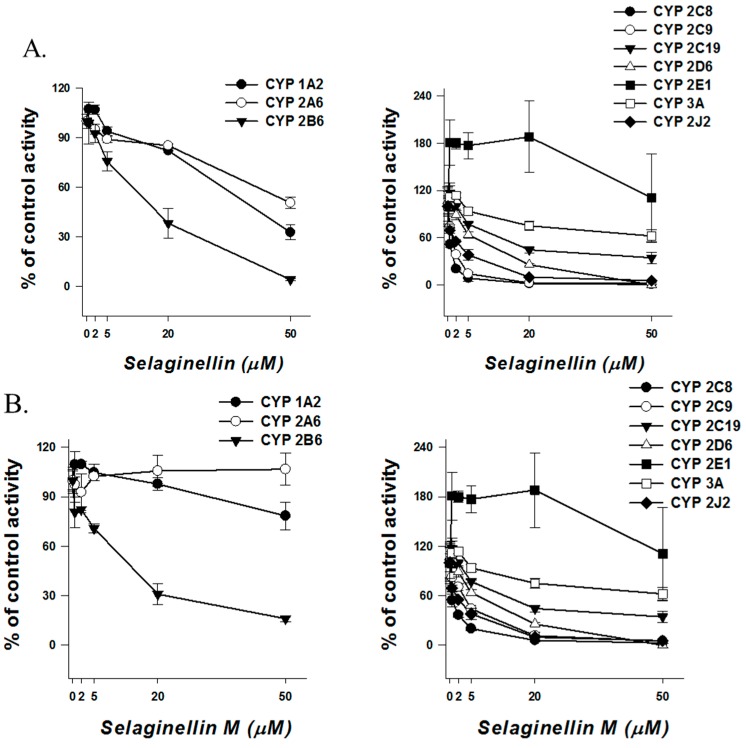

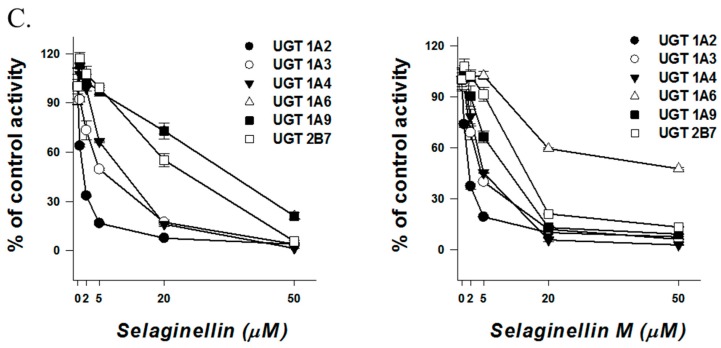
Inhibitory effects of selaginellin and selaginellin M against ten cytochromes P450 (**A**,**B**) and six uridine 5′-diphosphoglucuronosyltransferase enzymes (**C**). The activity is expressed as the percentage of the control activity. The data are shown as mean ± S.D. (*n* = 3).

In the present study, we investigated the inhibitory effect of two selaginellins against ten cytochrome P450 isoforms and six UGT isoforms using human liver microsomes (Figure 2). The results showed that selaginellin inhibited most of the P450 and UGT isoforms tested in a concentration-dependent manner. The inhibitory potential of selaginellins is categorized into high (IC_50_ < 1 μM), medium (1 μM < IC_50_ < 10 μM), and low (IC_50_ > 10 μM) classes based on Krippendorff’s criteria [24].

Selaginellin and selaginellin M had high inhibitory potential for CYP2C8-mediated amodiaquine *O*-demethylation (Table 1), respectively, indicating that herbal drugs containing selaginellins may be used carefully with drugs metabolized by CYP2C8, such as anti-cancer drugs (paclitaxel and sorafenib), antidiabetics (repaglinide), and diuretics (torsemide) in order to avoid drug interactions [25]. The inhibitory potential of these two selaginellins on CYP2C8 (IC_50_ < 1 μM) were lower than that of troglitazone (IC_50_ = 2.3 μM [26]) and quercetin (IC_50_ = 7.2 μM [27]). Their inhibitory potentials, however, were less potent than montelukast, an strong CYP2C8 inhibitor (IC_50_ = 0.019 μM [28]).

Two selaginellins also showed medium inhibitory potential on CYP2C9-catalyzed tolbutamide hydroxylation, CYP2J2-catalyzed astemizole *O*-demethylation, UGT1A1-catalyzed SN-38 glucuronidation, and UGT1A3-catalyzed chenodeoxycholic acid glucuronidation activities (IC_50_ < 5 μM). CYP2C8, CYP2C9, and CYP2J2 metabolize approximately 4.7, 12.8, and 3% of clinically used drugs (*n* = 248), respectively [29]. UGT1A1 also metabolizes approximately 17.3% of drugs (*n* = 237) which have glucuronidation as a clearance mechanism [30,31]. Therefore, the inhibitory effect of selaginellins might be important for producing potential herb–drug interaction with drugs which undergo CPY2C8, CYP2C9, CYP2J2, and UGT1A1-mediated biotransformation; such drugs include glipizide, irinotecan, losartan, paclitaxel, tolbutamide, and warfarin [32].

The effects on CYP1A1, CYP2A6, CYP2E1, and UGT1A6 activities were assumed to be a negligible (IC_50_ > 25 μM) (Table 1). These findings suggest that clinical interactions between these compounds and CYP1A1, CYP2A6, CYP2E1, or UGT1A6 would not occur.

*Selaginella tamariscina* (Beauv.) Spring has been used for centuries as a Traditional Chinese Medicine to treat various human diseases, including inflammation, human cancer, and hyperglycemia [33]. Therefore, it might be used with anticancer or antidiabetic drugs which are metabolized by CYP2C8 (paclitaxel), CYP2C9 (tolbutamide), or UGT1A1 (irinotecan) [32]. Selaginellins should be used carefully with these drugs to avoid drug interactions in cancer and diabetic patients.

## 3. Material and Methods

### 3.1. Reagents

Alamethicin, β-Nicotinamide adenine dinucleotide phosphate (NADP^+^), chenodeoxycholic acid, trifluoperazine, *N*-acetylserotonin, mycophenolic acid, naloxone, naloxone-β-d-glucuronide, uridine 5′-diphosphoglucuronic acid (UDPGA), glucose-6-phosphate (G6P), glucose-6-phosphate dehydrogenase (G6PDH), terfenadine (internal standard (IS) for P450 assay), estrone-β-d-glucuronide (IS for UGT assay), phenacetin, dextromethorphan, coumarin, chlorzoxazone, bupropion, astemizole, amodiaquine, acetaminophen, hydroxybupropion, hydroxycoumarin, hydroxychlorzoxazone, and *N*-desethylamodiaquine were purchased from Sigma-Aldrich (St. Louis, MO, USA). Tolbutamide, omeprazole, midazolam, dextrorphan, 1′-hydroxymidazolam, hydroxyomeprazole, hydroxytolbutamide, *N*-acetylserotonin-β-d-glucuronide, chenodeoxycholic acid-24-acyl-β-glucuronide, mycophenolic acid-β-d-glucuronide, SN-38, and SN-38-glucuronide were obtained from Toronto research Chemicals (Toronto, ON, Canada). Solvents were LC-MS grade (Fisher Scientific Co., Pittsburgh, PA, USA) and the other chemicals were of the highest quality available. Pooled human liver microsomes (HLMs, H2630, mixed gender) were purchased from XenoTech (Lenexa, KS, USA). Selaginellins: Selaginellin and selaginellin M were isolated from *Selaginella tamariscina* (Beauv.) which was collected at Hon Ba Nature Reserve, Khanh Hoa province, Vietnam. The two compounds were purified and examined by HPLC to get 95% purity. Their chemical structures were identified by analyzing their NMR data which were in good agreement with those published in a previous report [8].

### 3.2. Microsomal Incubation

#### 3.2.1. Inhibitory Effects of Selaginellins on P450 Activity

The inhibitory effects of two selaginellins on the metabolism of ten P450 probe substrates were evaluated using previously reported method with minor modification [27]. Phenacetin *O*-deethylase, coumarin 7-hydroxylase, bupropion 4-hydroxylase, amodiaquine *N*-deethylase, tolbutamide 4-hydroxylase, omeprazole 5-hydroxylase, dextromethorphan *O*-demethylase, chlorzoxazone 6-hydroxylase and midazolam 1′-hydroxylase activities were determined as probe activities for CYP1A2, CYP2A6, CYP2B6, CYP2C8, CYP2C9, CYP2C19, CYP2D6, CYP2E1 and CYP3A2, respectively, using substrate cocktail incubation and tandem mass spectrometry (Table 2). Selaginellins were dissolved in methanol. The final concentrations of organic solvent (methanol) for the cocktail incubation conditions in all experiments were 1.0% (*v*/*v*). In brief, the incubation mixtures containing pooled HLMs (0.25 mg/mL, H2630, Xenotech), P450 probe substrate cocktail, and inhibitor (0, 0.5, 2, 5, 20 and 50 μM) were preincubated at 37 °C for 5 min. The reaction was initiated by adding of the NADPH generating system (3.3 mM G6P, 1.3 mM β-NADP+, 3.3 mM MgCl_2_, and 1 unit/mL G6PDH) followed by incubation for 15 min at 37 °C. Next, each incubation was stopped by addition of 50 μL ice-cold acetonitrile containing terfenadine (IS). After mixing and centrifugation, aliquots were analyzed by liquid chromatography-tandem mass spectrometry (LC-MS/MS) as previous described [27,34]. The CYP2J2 inhibitory effects of two selaginellins were also evaluated in pooled HLMs using previously reported method [35,36]. In brief, the incubation reaction mixtures contained 0.25 mg/mL HLMs, astemizole (1 μM) and inhibitor (0.5–50 μM) in 0.1 mM phosphate buffer (pH 7.4). The reaction was initiated by the addition of NADPH-generating system and further incubated for 15 min. After reaction termination with cold acetonitrile containing 5 ng/mL terfenadine and centrifugation, aliquots were injected into a liquid chromatography-tandem mass spectrometry system (LC-MS/MS) as described previously [35] (Table 2). All microsomal incubations were performed in triplicate.

#### 3.2.2. Inhibitory Effects of Selaginellins on UGT Activity

The inhibitory effects of two selaginellins on the metabolism of six UGT probe substrates were evaluated using previously reported method with minor modification [34]. In brief, HLMs (0.25 mg/mL) were activated by incubation in the presence of alamethicin (25 μg/mL) for 15 min on ice. After the addition of UGT isoform-selective substrates and selaginellins (0, 0.5, 2, 5, 20 and 50 μM) the final concentrations of organic solvent (methanol) for the cocktail incubation conditions were 1.0% (*v*/*v*). The incubation reaction mixtures were pre-incubated for 5 min. The reaction was initiated by the addition of 5 mM UDPGA and further incubated for 60 min. All reactions were terminated by adding ice-cold acetonitrile containing estrone glucuronide (IS). After mixing and centrifugation, aliquots were injected into a LC-MS/MS as described previously [34] (Table 2). All microsomal incubations were performed in triplicate.

### 3.3. Data Analysis

The IC_50_ values (concentration of the inhibitor causing 50% inhibition of the original enzyme activity) were calculated using WinNonlin software (Pharsight, Mountain View, CA, USA): percentage of control activity = 100 × [1 − (I/(I + IC_50_))], where I is the inhibitor concentration, and IC_50_ is the inflection point on the curve [34].

## Figures and Tables

**Table 1:** Inhibitory effects of selaginellin and selaginellin M against ten cytochrome P450 (P450) and six uridine 5′-diphosphoglucuronosyl transferase (UGT) isoforms.

Compound	IC_50_ (μM)															
	P450 Isoforms										UGT Isoforms					
	1A2	2A6	2B6	2C8	2C9	2C19	2D6	2E1	2J2	3A	1A1	1A3	1A4	1A6	1A9	2B7
Selaginellin	36.4	>50	10.7	0.5	1.2	10.0	5.8	38.5	0.8	11.7	1.0	4.7	6.6	25.3	8.7	15.6
Selaginellin M	>50	>50	11.3	0.9	3.9	16.1	6.8	>50	2.7	>50	1.3	3.5	3.9	36.5	6.5	10.4

**Table 2:** Selected reaction monitoring (SRM) parameters for the metabolites of ten cytochrome P450 and six uridine 5′-diphosphoglucuronosyltransferase probe substrates.

Enzyme	Substrate	Concentration (μM)	Metabolite	Transition (*m*/*z*)	Collision Energy (eV)	Polarity *
CYP1A2	Phenacetin	100	Acetaminophen	152 > 110	25	ESI^+^
CYP2A6	Coumarin	5.0	Hydroxycoumarin	163 > 107	17	ESI^+^
CYP2B6	Bupropion	50	Hydroxybupropion	256 > 238	20	ESI^+^
CYP2C8	Amodiaquine	1.0	*N*-Desethylamodiaquine	328 > 283	17	ESI^+^
CYP2C9	Tolbutamide	100	Hydroxytolbutamide	287 > 89	42	ESI^+^
CYP2C19	Omeprazole	20	Hydroxyomeprazole	362 > 214	10	ESI^+^
CYP2D6	Dextromethorphan	5.0	Dextrorphan	258 > 157	35	ESI^+^
CYP2E1	Chlorzoxazone	50	Hydroxychlorzoxazone	184 > 120	15	ESI^−^
CYP2J2	Astemizole	1.0	*O*-Desmethyl astemizole	445 > 204	35	ESI^+^
CYP3A	Midazolam	5.0	Hydroxymidazolam	342 > 203	25	ESI^+^
UGT1A1	SN-38	0.5	SN-38-glucuronide	569 > 393	30	ESI^+^
UGT1A3	Chenodeoxycholic acid	2.0	Chenodeoxycholic acid glucuronide	567 > 391	35	ESI^−^
UGT1A4	Trifluoperazine	0.5	Trifluoperazine glucuronide	584 > 408	25	ESI^+^
UGT1A6	*N*-Acetylserotonin	1.0	*N*-Acetylserotonin glucuronide	395 > 219	15	ESI^+^
UGT1A9	Mycophenolic acid	0.2	Mycophenolic acid glucuronide	495 > 319	20	ESI^−^
UGT2B7	Naloxone	1.0	Naloxone glucuronide	504 > 310	30	ESI^+^

* Electrospray ionization in positive mode (ESI^+^) and negative mode (ESI^−^).
